# Performance Evaluation and Calibration of Electromagnetic Field (EMF) Area Monitors Using a Multi-Wire Transverse Electromagnetic (MWTEM) Transmission Line

**DOI:** 10.3390/s25092920

**Published:** 2025-05-05

**Authors:** Renzo Azaro, Roberto Franchelli, Alessandro Gandolfo

**Affiliations:** 1 EMC S.r.l., Erzelli Science and Technology Park, Via Sant’Elia 242, 16152 Genova, Italy; 2 NARDA Safety Test Solutions S.r.l., Via Benessea 29/B, Cisano sul Neva, 17035 Savona, Italy; roberto.franchelli@narda-sts.it (R.F.); alessandro.gandolfo@narda-sts.it (A.G.)

**Keywords:** electromagnetic measurements, EM field probe calibration, exposure to electromagnetic fields, multi-wire TEM transmission line

## Abstract

The exposure levels generated by environmental electromagnetic field (EMF) sources can be measured and monitored by employing EMF area monitors. The operating spectrum of environmental EMF sources is not limited to high frequencies (*f* > 30 MHz) but also extends to low frequencies (*f* < 30 MHz), where sources associated, for example, with radio transmitters typically generate non-negligible field contributions. For this reason, professional EMF area monitors can be equipped with different field sensors, properly calibrated according to standardized procedures. Because low-frequency electric fields are very sensitive to environmental boundary conditions, equipping an EMF area monitor with electric field sensors, previously calibrated as stand-alone devices, can lead to measurement errors due to field perturbations introduced by the physical structure of the area monitor itself. This paper describes the activities carried out to assess the performance of an EMF area monitor in simulated realistic conditions and calibrate it in the 300 kHz–20 MHz frequency band. The activities were conducted using a multi-wire transverse electromagnetic (MWTEM) transmission line as a controlled electric field source, with dimensions suitable for exposure of the entire structure of the EMF area monitor. In view of using this approach to calibrate the area monitors as a whole instead of the individual sensors, the uniformity of the electric field generated by the available MWTEM transmission line was analyzed in detail both numerically and experimentally. Finally, the results of the evaluation and calibration of an area monitor are reported and discussed.

## 1. Introduction

**Figure 1:**
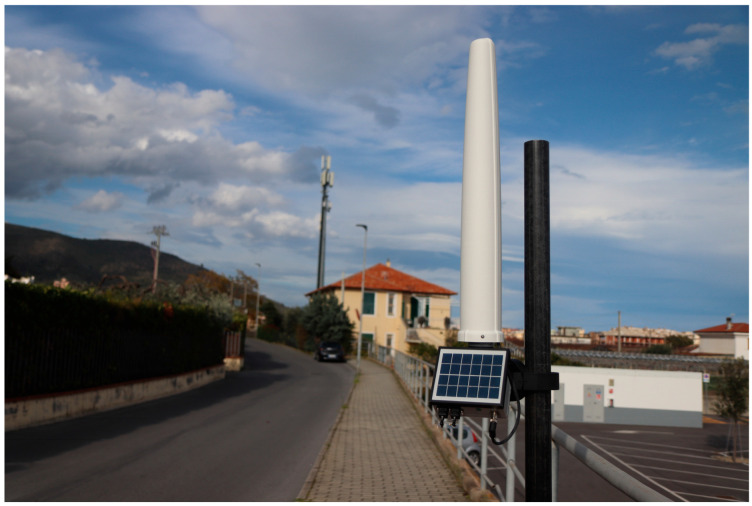
An example of a battery-operated EMF area monitor in a real application scenario (courtesy of NARDA Safety Test Solutions S.r.l.).

The rapid and continuous growth of wireless communications and data transmissions requires a great number of stations that generate non-negligible environmental electromagnetic fields. This significantly increases the density of radiating sources while at the same time increasing public concerns about exposure levels to the electromagnetic fields generated by the necessary systems. Similarly, the massive use of wireless technologies in a great variety of equipment and the pervasive distribution of electrical power networks implies a large number of intentional and non-intentional sources of electromagnetic energy being distributed in the environment and operating in a very wide frequency range, ranging from tens of Hz to tens of GHz. In this highly complex electromagnetic scenario, exposure to electromagnetic fields is a topic of great importance, especially for people living near infrastructures recognized as EMF sources. Local authorities and government agencies have an interest in the real-time, continuous monitoring of the exposure levels generated by different sources to ensure that nearby areas comply with applicable national or international standards [1,2,3,4]. Typical areas of concern are schools, hospitals, and residential and public areas, not to mention exposure in the workplace. Classical electromagnetic field measurement instruments, despite their optimized performance and accuracy, are not suitable for this kind of task as they generally require the presence of an operator; in addition, due to limited autonomous power supplies and a lack of weatherproofing, they are not suitable for long-term or continuous unattended outdoor monitoring. These limitations have led to the development of EMF area monitors, i.e., electromagnetic field measurement systems that can autonomously measure field levels and record and transmit data. EMF area monitors are typically battery-operated and equipped with solar panels for an independent power supply. In general, an EMF area monitor can be fitted with a set of field probes covering all parts of the spectrum of interest for a particular site to be monitored. An example of an EMF area monitor in a real application scenario is shown in Figure 1.

By using an EMF area monitor, the measurements taken with a certain time step can be read remotely at any time or automatically sent at preset intervals to a remote PC or a data server via the mobile network. Alarms can be automatically sent to selected recipients if preset limits are exceeded. When measurements are required from several positions, an effective solution is to implement a geographically distributed network of EMF area monitors that can continuously detect exposure levels, present the results to the public in an easily accessible format, and compare the results with the applicable standards [1,2,3,4]. Several researchers, from both academia and industry, have investigated ways of satisfying the requirements set by the International Telecommunication Union (ITU) [5] for the monitoring of environmental electromagnetic fields. Various studies based on the integration of field probes with wireless sensor networks (WSNs) [6,7,8] or with radio interfaces based on the GSM/GPRS standard [9,10,11,12,13,14] can be found in the literature. Recently, as described in [15], extensive geographical monitoring has been experimented by integrating an area monitor inside a car. The constant and current interest in the topic is demonstrated by the publication of scientific and technical papers, even in recent years [16,17,18,19,20]. In this framework, considering that an EMF area monitor is a more complex measuring system than a classical EMF probe and considering that it can operate in scenarios with different electrical boundary conditions (e.g., with or without cabling and a power supply unit for connection to the mains), researchers have studied the device’s behavior in different simulated environments [21].

In this paper, a description of the architecture of a typical EMF area monitor is followed by a presentation of the activities carried out in order to calibrate it as a whole system and validate its behavior. To this end, the electric field generated by a multi-wire TEM (MWTEM) transmission line was analyzed in detail both numerically and experimentally in order to evaluate its capability to generate controlled electric field distributions that are sufficiently uniform for the exposure of the entire structure of the EMF area monitor for frequencies up to 30 MHz.

In most cases, sources capable of generating non-negligible field contributions are associated with mobile phone systems or radio transmitters operating at frequencies higher than 30 MHz. However, in certain cases—often involving high emission levels—high-power RF sources operating at frequencies below 30 MHz are used for long-distance communication transmitters (e.g., for naval or military communications) or for industrial, scientific, and medical (ISM) applications, authorized by the ITU, such as dielectric heating, plastic welding, food processing, and short-wave diathermy.

## 2. The Structure of an EMF Area Monitor

**Figure 2:**
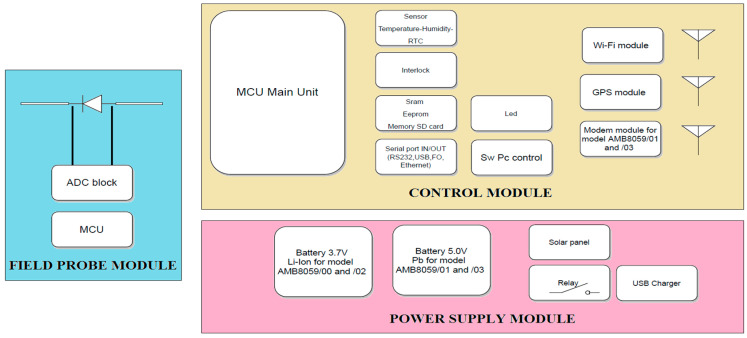
The structure and the main components of an EMF area monitor.

Figure 2 presents the general structure of the AMB-8059 area monitor. Three orthogonal broadband probes in the field probe module measure the field amplitude, and the resulting signal is converted by the analog to digital converter (ADC) and sent to the micro-controller unit (MCU). The area monitor, managed by the control module, is able to autonomously perform, acquire, memorize, and transmit measurements. The area monitor is equipped with a set of radio interfaces (Wi-Fi, GSM/GPRS, LTE) necessary for remote control and data exchange. The power supply module optimizes power consumption for long-time autonomous operation by managing battery use and charging via solar panel or, if available, via a power supply unit connected to the mains. Due to its non-negligible structure and the use of cables, the presence of an area monitor can modify the field under measurement, causing errors in the measurement results. This issue is magnified for frequencies below 30 MHz, as analyzed in [22] for electric field measurement with a rod antenna. To solve this kind of problem, the AMB-8059 is equipped with a relay that automatically disconnects the electrical connection when not needed. For the same reason, to improve measurement accuracy, the calibration of the area monitor has to be reconsidered as a whole system, as typically the field probes are calibrated as stand-alone devices and the effects of integrating them within the area monitor have to be evaluated and, if necessary, taken into account.

## 3. Experimental Setup

To calibrate and evaluate the performance of an area monitor as a whole system, we need to generate a controlled and sufficiently uniform electric field in a volume suitable for hosting the entire structure of the device under calibration. At higher frequencies (*f* > 30 MHz), this is easily accomplished by means of standard radiating devices like simple dipoles, biconical, log-periodic, and horn antennas. The same approach cannot be used at lower frequencies (*f* < 30 MHz), as in this frequency range, the physical size of dipolar antennas becomes impractical, and in order to operate under realistic electromagnetic field conditions, it is necessary to stay far from the antenna itself. This implies an overly challenging test setup while requiring the generation of intense electromagnetic fields over a large area.

A possible approach for the generation of high field levels in a confined region while avoiding conventional antennas is based on the use of transverse electromagnetic (TEM) transmission lines that in the enclosed volume support a uniform and linearly polarized plane wave [23,24,25].

Each transmission line composed of two separate conductors for *f* < *f_C_*_1_ can support the propagation of the TEM field configuration, while for *f* > *f_C_*_1_, other field configurations associated with high-order modes (*f_C_*_1_ being the cut-off frequency of the first high-order mode) can occur and heavily modify the field configuration. The main properties of a TEM wave are that the electric and magnetic fields are perpendicular to each other and both are transverse to the wave direction of propagation. This type of wave, known as a plane wave, is also present in the far field of an antenna radiating in free space. Coaxial lines, parallel plate lines, TEM cells, and similar structures (all characterized by the presence of two separate conductors or groups of conductors) can support this field configuration. For this reason, they are widely used to generate uniform fields for electromagnetic compatibility testing [26,27] and calibration. Wire array cells belong to this category, and thus, the multi-wire TEM line has been evaluated and validated for calibration activities.

Because classical TEM cells generally have small dimensions and a closed structure, it is very difficult to insert an entire area monitor inside their useful volume. They are therefore a good solution for calibrating smaller instruments like classical field probes, but impractical for equipment as large as an area monitor.

**Figure 3:**
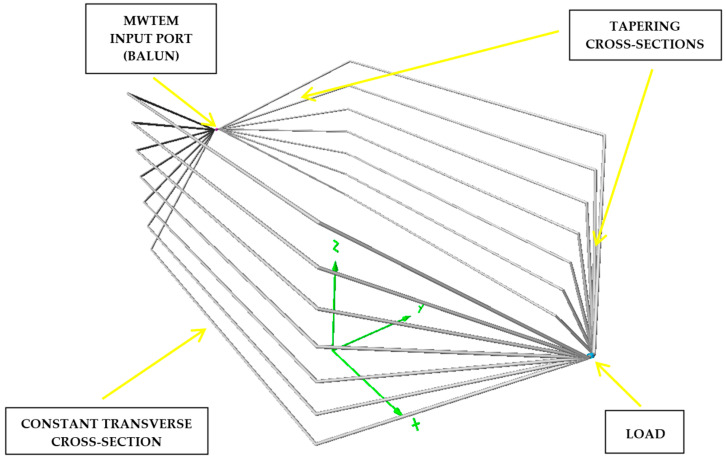
The geometry of the multi-wire TEM (MWTEM) transmission line.

An adequately sized alternative structure, composed of wire arrays and capable of supporting a TEM mode, is the multi-wire TEM (MWTEM) transmission line [28]. This device is less popular than TEM cells and, to the best of the authors’ knowledge, has never been used for the calibration and validation of field probes. The structure of the MWTEM line used for the activities described in this paper is shown in Figure 3, where three different sections can be observed: a central uniform multi-conductor transmission line enclosing the volume used for testing, and two lateral tapered transmission lines. At one end (the input port), the MWTEM line is equipped with a balun/matching network (50 Ω/200 Ω) and at the other end with a 200 Ω dummy load. The balun is necessary as no metallic element of the line supporting the TEM wave is connected to a constant potential (the device operates in balanced mode). The MWTEM line is equipped with a nominal 200 Ω load as this matches its characteristic impedance; according to transmission line theory, this is the optimal choice for minimizing reflections from the load and reducing standing wave phenomena along the line in the higher frequency band.

**Figure 4:**
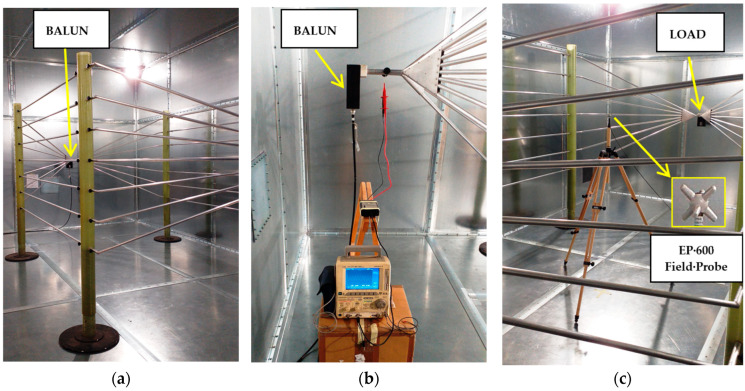
Experimental assessment of the electric field distribution generated by the MWTEM transmission line: (**a**) the line inside a shielded room; (**b**) measurement of the voltage amplitudes at the balanced input port; (**c**) electric field amplitude measurements with the EP600 probe in the central position of the line.

As shown in Figure 3, instead of metallic plates (as in classical TEM structures, e.g., the parallel plate line [26]), the two halves of the line are composed of an array of seven aluminum pipes (2 cm in diameter). These pipes are parallel in the central section and taper between the central section and the two ends. The MWTEM line used for experiments is pictured in Figure 4. In particular, Figure 4b,c show the two ends of the line with the balun and the load, respectively.

The use of a TEM cell composed of wire arrays instead of planar elements greatly complexifies the electromagnetic problem to be solved in order to find the electrical parameters of the device and accurately analyze the uniformity of the generated electric field. Because no analytical formula is available for the field values and characteristic impedance, a simulation software has been used to obtain a detailed and accurate characterization of the device. The results of the characterization by numerical simulation are reported and discussed in Section 4.

An outline of the theory of MWTEM cells is reported in Appendix A. Starting from the description of the TEM mode field components existing in the basic structure of the parallel plate line, an approximated theory of the TEM mode valid for an MWTEM in a quasi-static condition is reported. The relationships obtained according to the quasi-static model can be profitably employed for the design of a generic N-wire MWTEM line in terms of transversal electric field components and line impedance.

The MWTEM transmission line is an open structure, so when driven by radiofrequency power, it can radiate non-negligible fields in the surrounding area. During the experimental activities, it was driven using a 75 W power amplifier, then placed inside a shielded chamber to minimize interference with sensitive equipment in the nearby area. The technical characteristics of all equipment used during the experiments are summarized in Table 1.

## 4. Numerical and Experimental Assessment of the Electric Field Generated by the MWTEM Transmission Line

Due to the absence of detailed technical documentation about the spatial distribution of the field generated by the MWTEM transmission line, before its use for area monitor calibration and evaluation, the distribution and uniformity of the electric field generated inside its useful volume was analyzed by means of both numerical simulations and measurements. For simulations, at the input port of the line (between the balun and the tapered section), the same set of applied peak-to-peak voltage amplitudes *V_p-p_* used during the experimental activities was considered. As shown in Figure 4b, the voltages were detected by means of a suitable differential probe connected to the balanced input port of the MWTEM transmission line and with the help of a digital oscilloscope. The numerical simulations were performed with a method of moment (MoM) algorithm by using a model of the MWTEM transmission line developed according to the guidelines of the employed software, i.e., the Numerical Electromagnetics Code (NEC). As described in detail in [29,30,31], NEC uses both an electric field integral equation (EFIE) and a magnetic field integral equation (MFIE) to model the electromagnetic response of systems composed of conductive wires and surfaces. Each formulation has advantages for specific cases: EFIE is well suited for small-volume thin-wire structures, while MFIE works well for large surfaces. Assuming a time-harmonic dependance $e^{j\omega t}$, under the assumption of thin wires, the electric field integral equation (EFIE) can be written as [29,30,31]:

(1) $$-\hat{n}\left(\vec{r}\right)\times {\vec{E}}^{I}\left(\vec{r}\right)=-\frac{j\eta }{4\pi k}\hat{n}\left(\vec{r}\right)\times \int_{L}I\left(s^{\prime }\right)\left(k^{2}{\hat{s}}^{\prime }-\nabla \frac{\partial }{\partial s^{\prime }}\right)g\left(\vec{r},\vec{r}\prime \right)ds\prime$$

where:

gr→,r→′ =e−jkr→−r→′r→−r→′

${\vec{E}}^{I}$ is an incident electric field;

$k=\omega \sqrt{\mu_{0}\varepsilon_{0}}$, $\eta =\sqrt{\mu_{0}/\varepsilon_{0}}$;

$\vec{r}$ is the observation point;

$\hat{n}\left(\vec{r}\right)$ is the unit normal vector of the surface at $\vec{r}$;

I is the current flowing in the wire having length *L;*

s is the distance parameter along a wire axis;

$\hat{s}$ is the unit vector tangent to wire axis.

The magnetic field integral equation (MFIE) can be written as:

(2) $$-\hat{n}\left({\vec{r}}_{0}\right)\times {\vec{H}}^{I}\left({\vec{r}}_{0}\right)=-\frac{1}{2}{\vec{J}}_{s}\left({\vec{r}}_{0}\right)+\frac{1}{4\pi }\int_{S}\hat{n}\left({\vec{r}}_{0}\right)\times \left[{\vec{J}}_{s}\left(\vec{r}\prime \right)\times \nabla \prime g\left({\vec{r}}_{0},\vec{r}\prime \right)\right]dA\prime$$

where:

${\vec{H}}^{I}$ is an incident magnetic field;

${\vec{J}}_{S}$ is a surface current density induced on a surface *S*;

${\vec{r}}_{0}$ is a surface point;

$\hat{n}\left({\vec{r}}_{0}\right)$ is the unit vector normal to *S* at ${\vec{r}}_{0}$.

The integral Equations (1) and (2) are solved numerically in NEC by the method of moments (MoM) [32]. A brief review of this method is provided in Appendix B.

The ${\vec{E}}_{tot}$ amplitudes were calculated for a discrete set of frequencies using the NEC implementation available in the 4NEC2 (V. 5.9.3) software package [31], considering a plane transversal to the line axis (x-axis) being placed in the center of the length of line (x = 0 m) and a plane parallel to the line axis (x-axis) and orthogonal to the y-axis (i.e., a longitudinal plane). The geometry of the MWTEM transmission line and the axes of the coordinate system are shown in Figure 3. The main parameters of the simulations and the results obtained in the central position of the transversal and longitudinal sections (x = 0, y = 0, z = 1.55 m) are summarized in Table 2 and Table 3. In Table 2, for each frequency value considered, the following data are reported: the peak-to-peak voltage values *V_p-p_* applied at the balanced input port of the MWTEM line, the electric field amplitudes measured with an EP600 field probe, the field value calculated with the formula E→=Vrmsd (where d = 2.32 m is the distance between the two halves of the MWTEM line in the central section), and the simulated field values obtained assuming a constant 200 Ω load.

For a more accurate characterization of the device, the complex values of the load impedance (composed of non-inductive high-power resistances) were measured with a network analyzer, because, as expected, they differed markedly from the nominal value (200 Ω) because of the non-ideal behavior of the lumped components making up the load. Table 3 reports, for each frequency, the measured complex load impedance values and the simulated electric field amplitudes (obtained with the measured load impedances). To evaluate the effects and take them into account, simulations were also performed with the MWTEM line being placed inside a shielded chamber having the characteristics reported in Table 1 and pictured in Figure 4.

**Figure 5:**
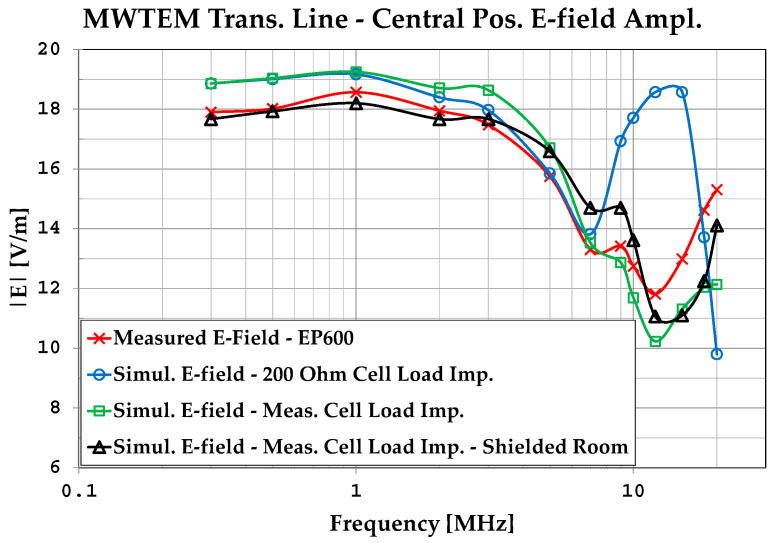
Simulated and measured E-field amplitude in the central position of the MWTEM transmission line.

An initial observation from Table 2 is that the formula E→=Vrmsd always overestimates the actual field values. In Figure 5, the results obtained with the MoM algorithm are graphed and compared with the measured values. By analyzing the plots in Figure 5, we can observe that the simulations assuming a constant resistive load (200 Ω) provide a good representation of the field amplitude up to about 7 MHz, while for higher frequencies, it is necessary to use the actual load impedance.

**Figure 6:**
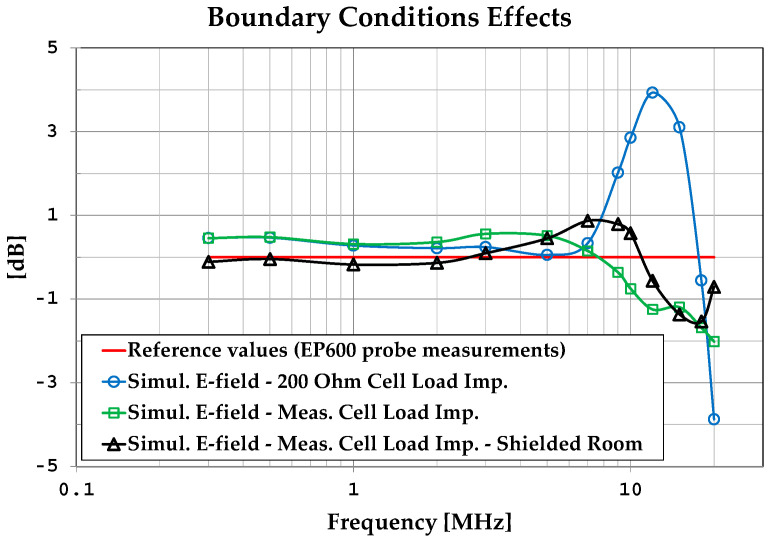
Effects of boundary conditions: MWTEM line load value (nominal 200 Ω, measured) and interaction with the shielded chamber.

**Figure 7:**
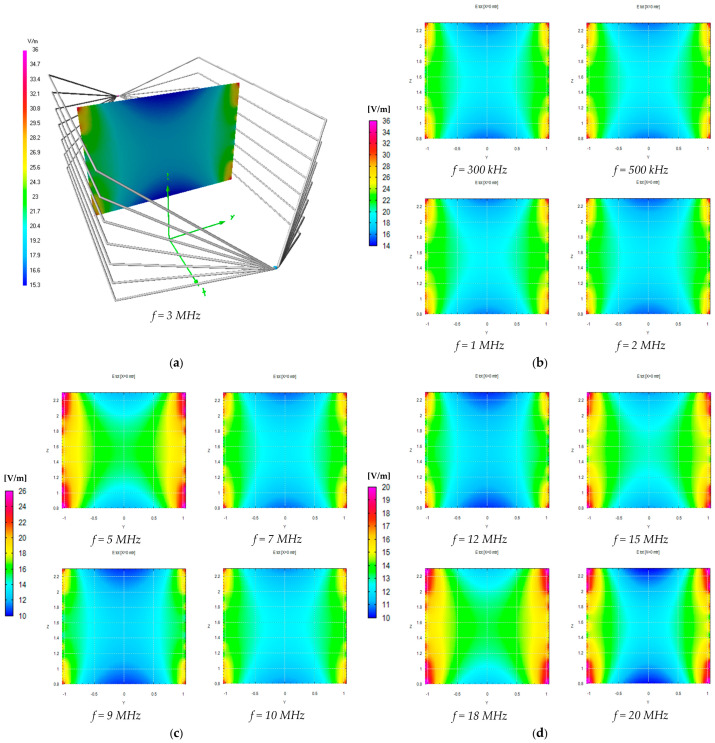
Numerical evaluation of the uniformity of the electric field generated inside the volume formed by the MWTEM transmission line: (**a**) geometry of the line and the transversal section considered for the simulations (results shown for *f* = 3 MHz); (**b**) results for *f* = 0.3 MHz, *f* = 0.5 MHz, *f* = 1 MHz, and *f* = 2 MHz; (**c**) results for *f* = 5 MHz, *f* = 7 MHz, *f* = 9 MHz, and *f* = 10 MHz; (**d**) results for *f* = 12 MHz, *f* = 15 MHz, *f* = 18 MHz, and *f* = 20 MHz.

**Figure 8:**
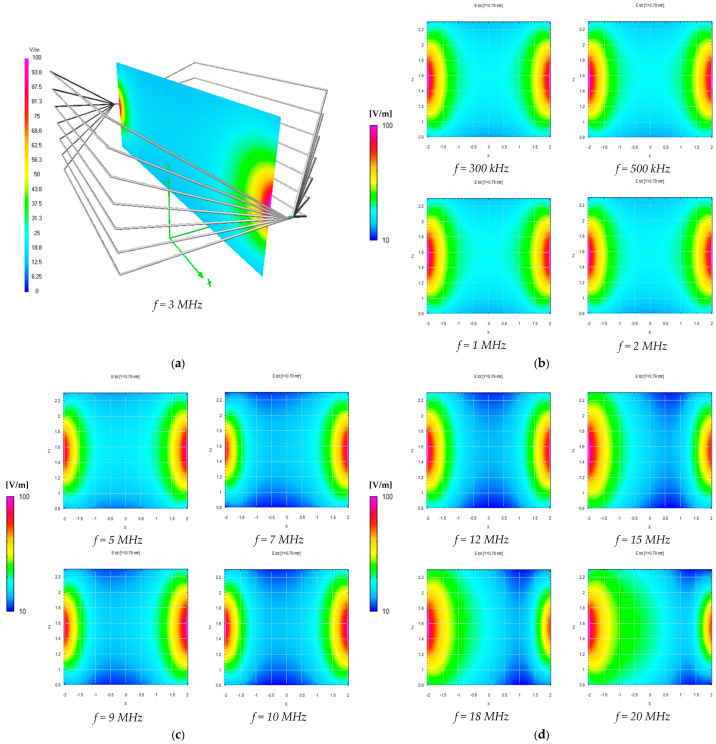
Numerical evaluation of the uniformity of the electric field generated inside the volume formed by the MWTEM transmission line: (**a**) geometry of the line and the longitudinal section considered for the simulations (results shown for *f* = 3 MHz); (**b**) results for *f* = 0.3 MHz, *f* = 0.5 MHz, *f* = 1 MHz, and *f* = 2 MHz; (**c**) results for *f* = 5 MHz, *f* = 7 MHz, *f* = 9 MHz, and *f* = 10 MHz; (**d**) results for *f* = 12 MHz, *f* = 15 MHz, *f* = 18 MHz, and *f* = 20 MHz.

**Figure 9:**
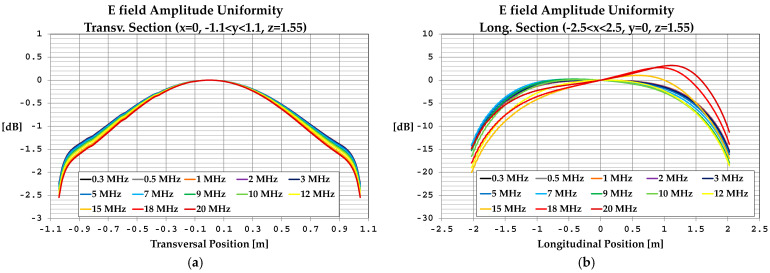
Numerical evaluation of the uniformity of the electric field generated inside the volume formed by the MWTEM transmission line: (**a**) field behavior along the transversal direction (x = 0, −1.1 < y < 1.1, z = 1.55 m); (**b**) field behavior along the longitudinal direction (−2.5 < x < 2.5, y = 0, z = 1.55 m).

We can also observe good accordance between measurements and simulations, in particular between the values measured with the EP600 probe and those obtained by simulations considering the measured load impedances and the effects of the shielded chamber. These results confirm the effectiveness of the model used for the simulations, in particular for the field uniformity analysis. The measurements taken with the EP600 probe can be assumed to be reliable as this device is very small, battery-operated, and connected to a PC with control software through a fiber optic cable, allowing for high measurement accuracy. Taking the field amplitude measured with the EP600 probe as a reference, Figure 6 shows the deviations observed with three different boundary conditions assumed during the simulations: the MWTEM line with the nominal load impedance (200 Ω), MWTEM line with the measured load impedance, and MWTEM line with the measured load impedance inside the shielded chamber. The plots in Figure 6 confirm the important role of the load and indicate that the use of the effective impedance values of the load greatly increases the accuracy of the calculated field values; in addition, the shielded chamber appears to have had an appreciable but limited effect. Excluding the configuration with nominal load impedance, the effects of the boundary conditions are evident for frequencies higher than about 8 MHz, where deviations of about 2 dB can be observed. For lower frequencies, the deviations are very small (about ± 0.5 dB); the limited effect of the shielding chamber confirms that the electric field is mainly confined within the useful volume of the MWTEM line, as occurs in static conditions. To evaluate the uniformity of the electric field generated by the MWTEM line, the results obtained in the two orthogonal planes were analyzed in detail. Figure 7b–d presents the plots of simulated $\left|{\vec{E}}_{tot}\right|$ values in the transversal plane, while the corresponding plots in the longitudinal plane can be observed in Figure 8b–d. The simulations were performed for the same set of frequency values considered during the experimental activities, listed in Table 2, with a step of 1.5 cm (along x, y and z) for the electric field calculation in both the transversal and the longitudinal planes. As can be observed in Figure 7 and Figure 8, in the central volume of the MWTEM transmission line, the electric field amplitude is sufficiently uniform. In more detail, the plots in Figure 9 show the difference (in dB) between the field amplitude in the central position (x = 0, y = 0, z = 1.55 m) and the values calculated moving along the y-direction (−1.1 < y < 1.1 m, Figure 9a) and along the x-direction (−2.5 < x < 2.5 m, Figure 9b).

By analyzing these data, it can be observed that the field in the central zone shows small and regular variations: about 1.5 dB for a position variation of ±0.9 m in the transversal plane and about 2 dB for a position variation of ±0.5 m in the longitudinal plane. Taking into account that in a TEM structure the electric field is almost transversal, its proper use is to host the area monitor with the axis lying in the transversal plane, where the electric field amplitude is sufficiently uniform in a spatial interval adequate to host the device under testing. The electric field generated for the area monitor calibration is in practice in the near-field region with respect to the field sources; then, to verify the existence in the volume formed by the MWTEM transmission line of a TEM field configuration, a detailed numerical analysis of the field components was also carried out. The analysis was performed on a transversal and a longitudinal cross-section, having the dimensions 1 × 1 m. The results, reported in detail in Appendix C, allowed us to verify that, according to the TEM configuration definition, the longitudinal components of both the electric field vector and the magnetic field vector are negligible if compared with the transversal components. In addition, it was also possible to confirm that the electric field vector is mainly directed along the transversal *y*-direction and the magnetic field along the transversal *z*-direction (i.e. the electric and the magnetic fields are mutually orthogonal).

The models of the experimental setup, in free space and inside the shielded chamber, followed the modeling guidelines of the NEC software [31]. The metallic enclosure of the shielded chamber was formed with wire grids according to the modelling concepts given in [33,34,35] for conductive surfaces. The use of the discretization criteria given in [31] (expressed in terms of the length of the segments composing the wires of the MWTEM structure, normalized to the wavelength) guarantees the high accuracy of the results. Some details about the models and the computational effort are summarized in Table 4. All of the simulations were carried out on a personal computer equipped with an Intel Core i7 CPU 2.10 GHz and with 8 GB of RAM.

## 5. Experimental Evaluation and Calibration of the EMF Area Monitor

After completing the assessment of the uniformity of the electric field generated in the central volume of the MWTEM transmission line using an EP600 field probe as a reference, the field generated by the MWTEM line was employed to evaluate and calibrate the AMB-8059 area monitor (provided by NARDA STS S.r.l., Cisano sul Neva, Savona, Italy) equipped with a solar panel and an EP-1B-02 wideband probe (frequency band 0.1–3000 MHz, amplitude range 0.2–200 V/m). This made it possible to evaluate the frequency behavior of the area monitor (equipped with the EP-1B-02 probe as a unique field sensor) and determine its correction factor (C.F.), defined, as in (3), at a given frequency and with a fixed electric field amplitude, as the ratio of the simulated electric field amplitude in the central position of the MWTEM line (assumed as the reference field level $E_{REF.}$) to the measurement ($E_{MEAS.}$) obtained with the area monitor placed with the EP-1B-02 probe in the same position:

(3) $$C.F.\;\left[dB\right]=20{Log}_{10}\left(\frac{E_{REF.}}{E_{MEAS.}}\right)$$

**Figure 10:**
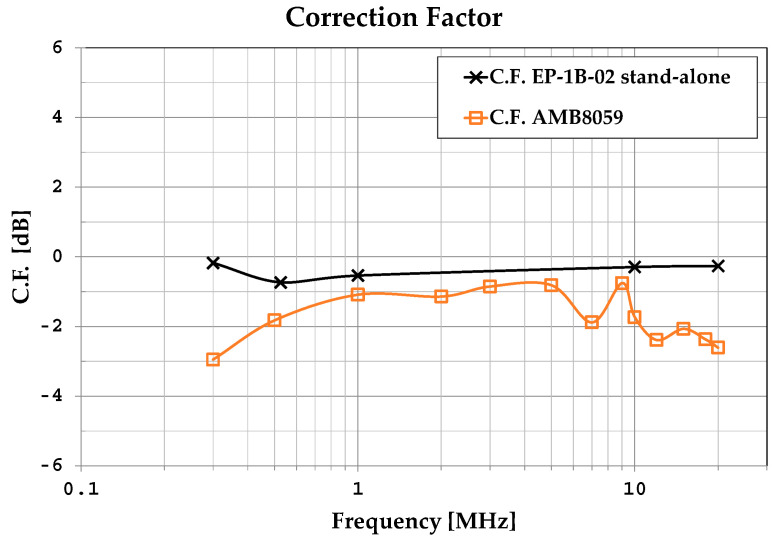
Comparison between the C.F. of the EP-1B-02 probe integrated into the area monitor and the C.F. of the EP-1B-02 probe as a stand-alone device.

In practice, during real on-site measurement activities, the correction factor has to be applied to the measured field to obtain the effective value of the field under measurement. This is performed manually, or automatically if the C.F. is stored in the area monitor’s control module. In Figure 10, the C.F. obtained for the area monitor as a whole instrument is compared with the correction factor of the EP-1B-02 probe calibrated as a stand-alone device. As expected, the frequency response of the field probe integrated into the area monitor is not the same as that of the probe alone; when integrated within the area monitor, the probe shows a less uniform frequency behavior and also proves to be a bit more sensitive (differences vary between 0.5 dB and 2.7 dB).

Knowing the C.F. makes it possible to improve measurement accuracy when a source with a known spectrum position is under measurement; the availability of a correction factor is of paramount importance in several practical cases where the area monitor is placed in the proximity of a known source deemed to be continuously monitored. When the spectral position of the source is known, the use of the precise C.F.—valid for the emission frequency of the source—allows for the optimum improvement of measurement accuracy, especially if the C.F. is available with a sufficiently small frequency step. In addition, when electromagnetic pollution is generated by unknown sources, the frequency behavior of the correction factor can be used to define a mean value to be applied to measurements in order to reduce uncertainty. Regarding different types of sources, we assume a source to be known if its emission frequency band is identified. For example, a source is considered to be known if it generates an exposure level at a site and is associated with a broadcasting system whose emission frequency is known either from the system owner or from preliminary spectral narrow-band measurements. Conversely, a source is considered unknown at a site where no information on its emission spectrum is available from the owner or from narrow-band measurements. In addition, knowing the emission frequency bands of multiple sources is ineffective if they all contribute to a non-negligible exposure level at the same site. In such cases, it is impossible to apply different C.F.s to measurements taken with wideband probes (such as those integrated into the area monitor) as they provide only a single measurement value, representing the combined effects of all sources. Therefore, when dealing with unknown sources, using a single C.F. value—such as the mean C.F.—is likely the best approach.

**Figure 11:**
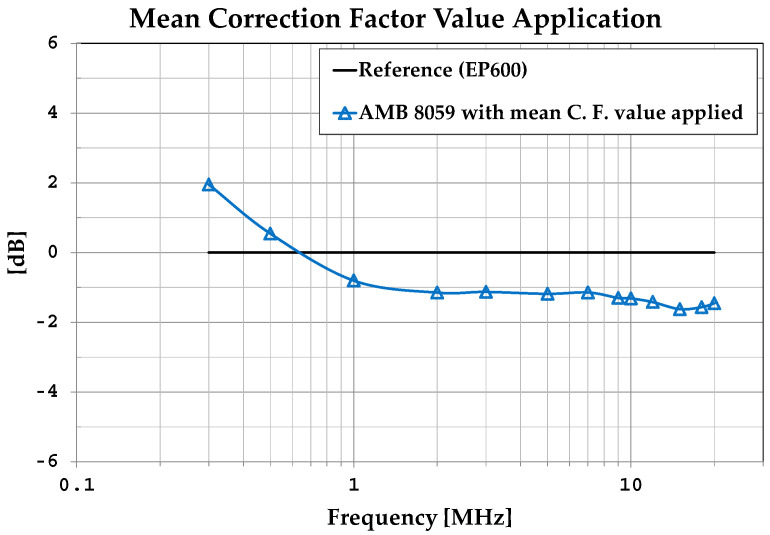
Effect of applying the mean C.F. value.

To verify the effectiveness of using the mean C.F. value, an electric field was generated in the MWTEM line and measured in its central position with the area monitor at the same frequencies considered for the calibration. The deviations of the measurements taken with the area monitor after the application of the mean C.F. value from the measurements taken under the same conditions with the EP600 probe (assumed as references) are shown in Figure 11. We can observe that the application of a mean constant C.F. for frequencies higher than 1 MHz makes the sensor’s frequency response uniform and implies errors of about 1 dB; for frequencies lower than 1 MHz, it implies higher yet still limited errors of around 2 dB.

Future activities will investigate the feasibility of specific correction factors associated with different ambient area configurations, e.g., those equipped with devices and cables for specific applications. Further analysis will also be performed to evaluate the uncertainty associated with the correction factor measurement procedure.

## 6. Conclusions

The electric field generated by an MWTEM transmission line was analyzed in detail, both numerically and experimentally, to evaluate its capability to generate for *f* < 30 MHz a controlled electric field sufficiently uniform for the exposure of the entire structure of an EMF area monitor. Typically, the field probe integrated in an area monitor is calibrated as a stand-alone device without taking into account possible effects on the probe response due to electromagnetic interaction with the area monitor structure and materials. To avoid such problems and improve measurement accuracy, the ambient area monitor was calibrated by exposing the whole structure of the device to the electric field generated by the previously characterized MWTEM line. A frequency-varying correction factor (C.F.) to be applied in the case of known field sources was determined, and the effectiveness of using a mean (constant) C.F. value in the case of unknown sources was evaluated.

## Figures and Tables

**Table 1:** Characteristics of equipment used for the experimental activities.

Equipment	Manufacturer	Model	Characteristics
Shielded Chamber	Beltech	-	Dimensions: 6.1 × 4.9 × 3.6 m
MWTEM	Comtest	-	7 + 7 wires balanced transm. line, 4.5 × 2.3 × 1.5 m, Z_0_ = 200 Ω, *f*_max_ = 20 MHz
MWTEM Balun	Comtest	-	Impedances levels: 50 Ω /200 Ω
MWTEM Load	Comtest	-	Impedance: 200 Ω (nominal value)
RF Signal Generator	Hewlett Packard	E4400B	Freq. band: 250 kHz–1 GHz
Power Amplifier	Amplifier Research	75A250	Freq. band: 10 kHz–250 MHz, P_max_ = 75 W
Digital Oscilloscope	Yokogawa	DL1620	*f*_max_ = 200 MHz
High Voltage Diff. Probe	Sapphire Instruments	SI-9010	*V*_max_ = ±7000 V, *f*_max_ = 70 MHz
Electric Field Probe	Narda STS	EP600	Freq. band: 100 kHz–9.25 GHz, 0.14–140 V/m
Network Analyzer	Agilent	E5061B	Freq. band: 5 Hz–3 GHz

**Table 2:** MWTEM transmission line—simulation parameters and results.

Freq. [MHz]	Applied Voltage [V_p-p_]	EP600 Meas. [V/m]	$\left|{\vec{E}}_{tot}\right|$ calc. ^1^ [V/m]	$\left|{\vec{E}}_{tot}\right|$ Simul. (Z_nom_ = 200 Ω) [V/m]
0.3	162.5	17.93	24.52	18.86
0.5	162.5	18.02	24.74	19.01
1	164.5	18.57	24.91	19.17
2	158.3	17.95	23.83	18.40
3	153.1	17.48	23.18	17.97
5	133.3	15.75	20.17	15.85
7	113.3	13.30	17.37	13.82
9	139.5	13.42	21.25	16.93
10	145.8	12.75	22.37	17.71
12	160.4	11.81	24.26	18.57
15	176.1	12.99	26.81	18.58
18	150.0	14.62	22.50	13.71
20	114.5	15.31	17.37	9.80

^1^ Value calculated with the formula E→=Vrmsd, where d = 2.32 m.

**Table 3:** MWTEM transmission line—effects of measured load impedance values and of a shielded chamber.

Freq. [MHz]	Z_meas_. [Ω]	$\left|{\vec{E}}_{tot}\right|$ Simul. [V/m]	$\left|{\vec{E}}_{tot}\right|$ Simul. (with Shield) [V/m]
0.3	201-j5	18.86	17.67
0.5	201-j8	19.04	17.93
1	200-j15	19.25	18.20
2	196-j30	18.71	17.63
3	189-j43	18.64	17.67
5	171-j58	16.71	16.59
7	153-j72	13.53	14.70
9	133-j79	12.87	14.74
10	124-j80	11.69	13.62
12	107-j80	10.23	11.07
15	86-j74	11.32	11.10
18	68-j64	12.04	12.25
20	58-j51	12.14	14.11

**Table 4:** NEC model parameters and simulation effort details.

NEC Model	Number of Segments	Simulation Time ^1^ [s]
MWTEM in free space	2100	30
MWTEM inside shielded chamber	9062	1743

^1^ Simulation time necessary for each frequency value.

**Figure A1:**
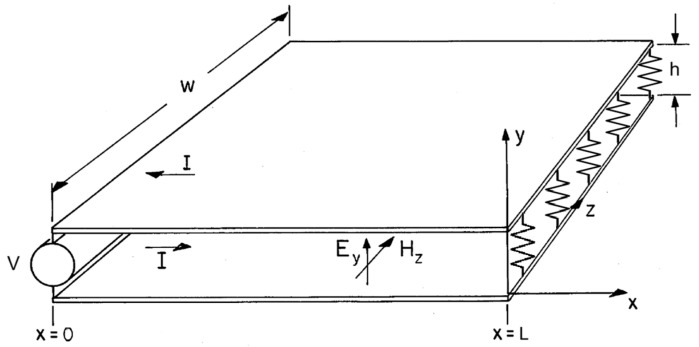
The parallel plate transmission line.

**Figure A2:**
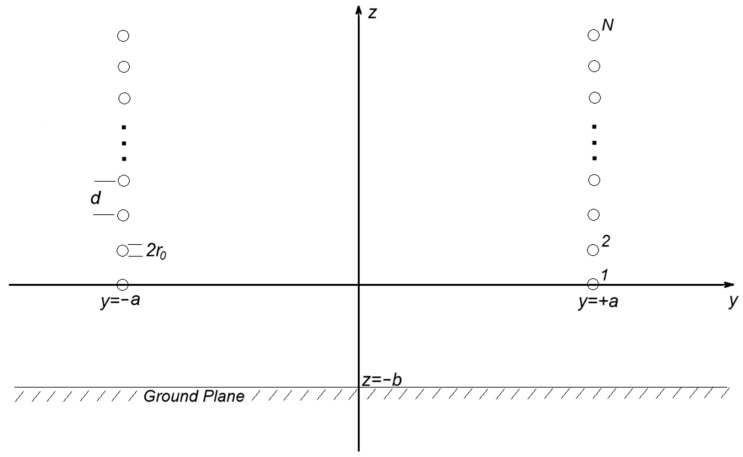
The transversal section of an N-wire MWTEM transmission line.

**Figure A3:**
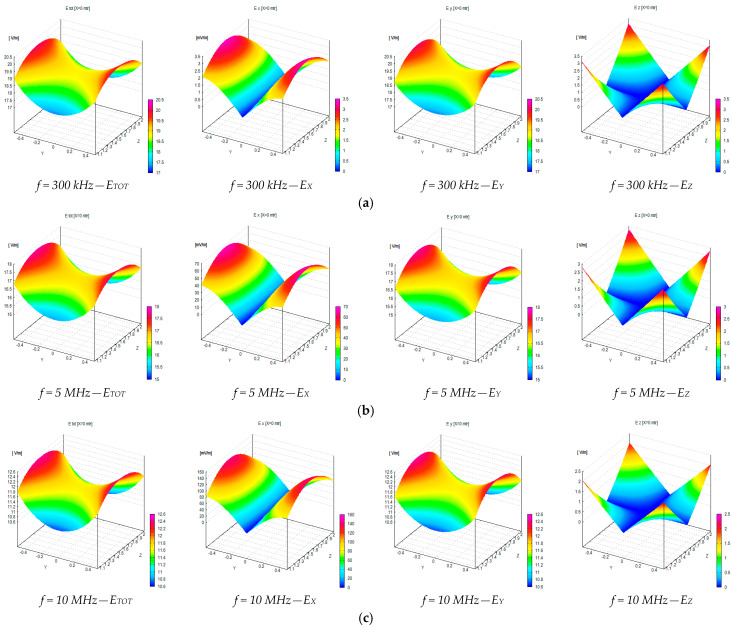

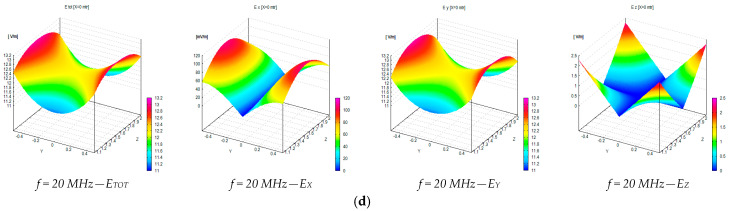
Numerical evaluation of the components (E_X_, E_Y_, E_Z_) of the electric field generated on the central transversal section inside the volume formed by the MWTEM transmission line: (**a**) *f* = 0.3 MHz, (**b**) *f* = 5 MHz, (**c**) *f* = 10 MHz, (**d**) *f* = 20 MHz.

**Figure A4:**
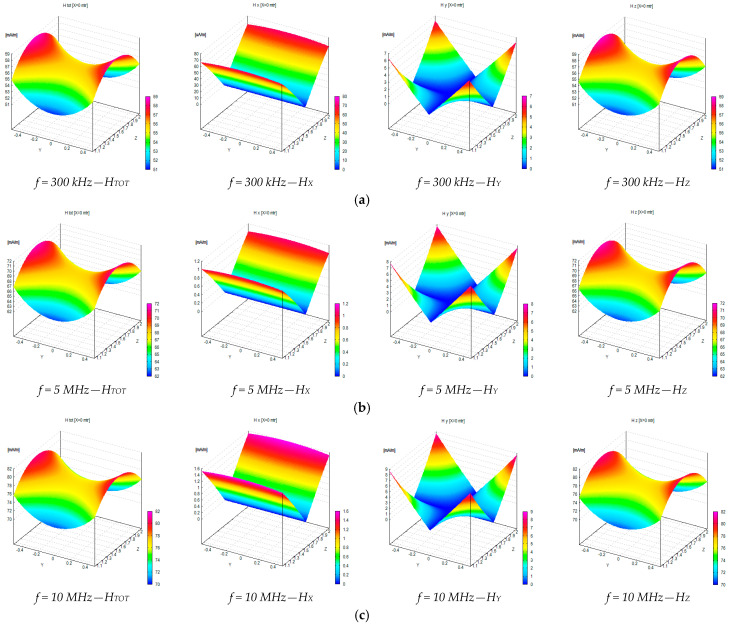

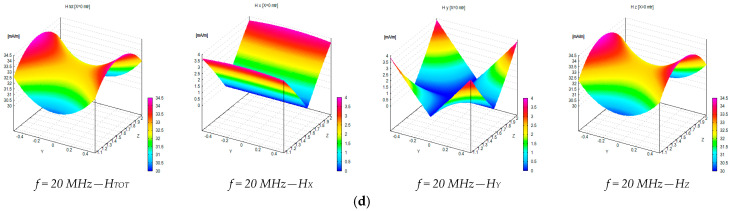
Numerical evaluation of the components (H_X_, H_Y_, H_Z_) of the magnetic field generated on the central transversal section inside the volume formed by the MWTEM transmission line: (**a**) *f* = 0.3 MHz, (**b**) *f* = 5 MHz, (**c**) *f* = 10 MHz, (**d**) *f* = 20 MHz.

**Figure A5:**
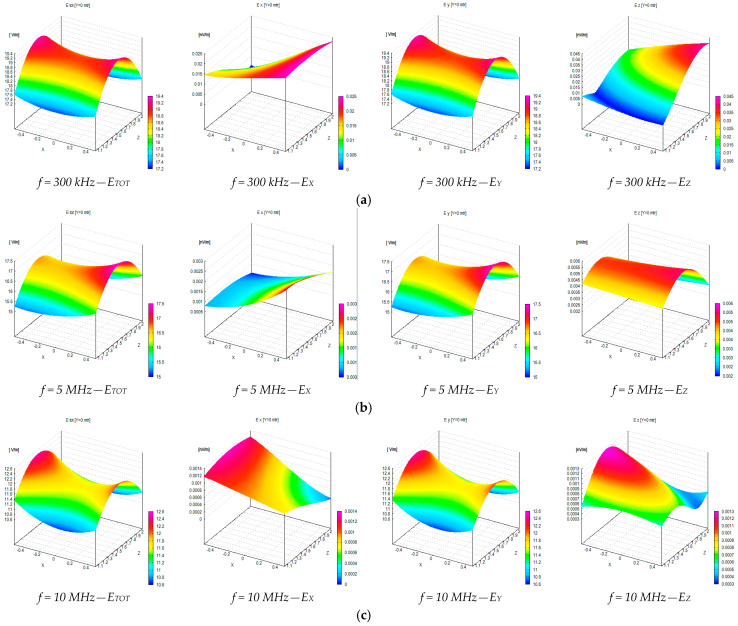

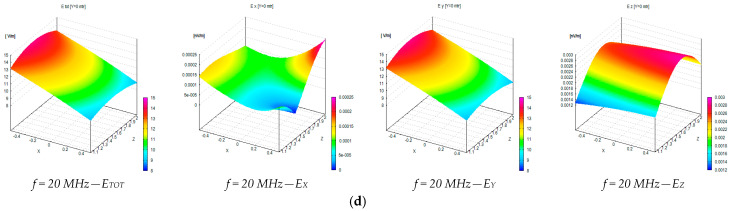
Numerical evaluation of the components (E_X_, E_Y_, E_Z_) of the electric field generated on the central longitudinal section inside the volume formed by the MWTEM transmission line: (**a**) *f* = 0.3 MHz, (**b**) *f* = 5 MHz, (**c**) *f* = 10 MHz, (**d**) *f* = 20 MHz.

**Figure A6:**
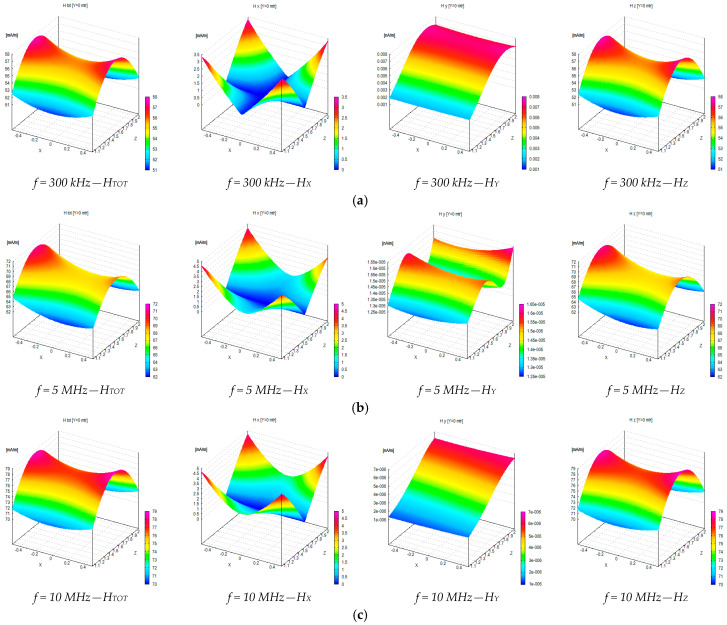

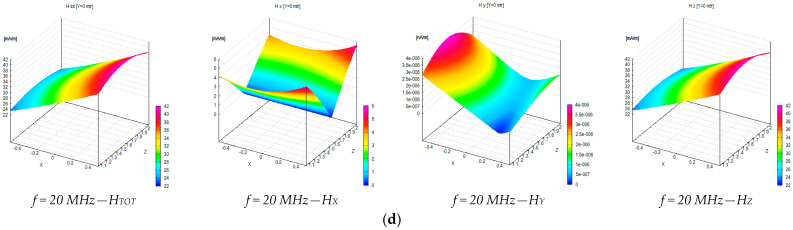
Numerical evaluation of the components (H_X_, H_Y_, H_Z_) of the magnetic field generated on the central longitudinal section inside the volume formed by the MWTEM transmission line: (**a**) *f* = 0.3 MHz, (**b**) *f* = 5 MHz, (**c**) *f* = 10 MHz, (**d**) *f* = 20 MHz.

**Figure A7:**
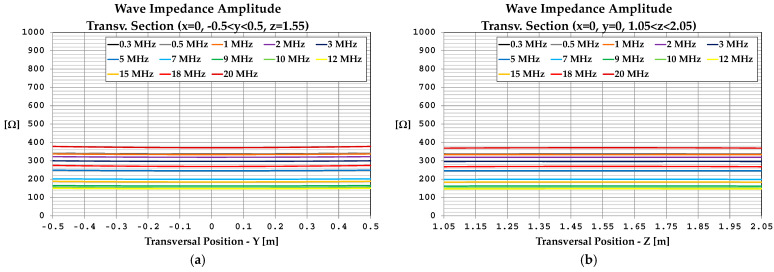
Numerical evaluation of the near field inside the volume formed by the MWTEM transmission line: (**a**) wave impedance along the transversal direction (x = 0, −0.5 < y < 0.5, z = 1.55 m), (**b**) wave impedance along the transversal direction (x = 0, y = 0, 1.05 < z < 2.05 m).

**Figure A8:**
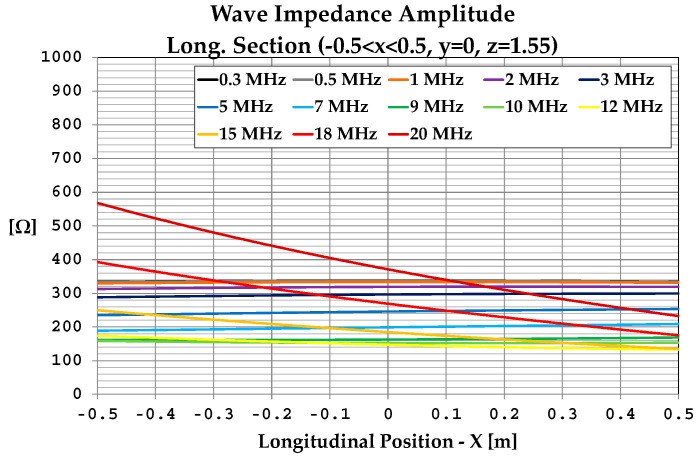
Numerical evaluation of the near field inside the volume formed by the MWTEM transmission line: wave impedance along the longitudinal direction (−0.5 < x < 0.5, y = 0, z = 1.55 m).

## Data Availability

Data are contained within the article.

## Abbreviations

The following abbreviations are used in this manuscript:

EMF	Electromagnetic field
HF	High frequency
LF	Low frequency
MWTEM	Multi-wire transverse electromagnetic
MoM	Method of moments

## Appendix A

The basic structure that can be employed to explain the operation of the MWTEM line from a theoretical point of view is the parallel plates transmission line. Because in practice it is relatively inexpensive and easy to construct, even for large products, the parallel plane line has long been used in electromagnetic compatibility activities for the susceptibility testing of electrical and electronic products and, in general, for the generation of a reference electromagnetic field. The geometry of a parallel plate transmission line is shown in Figure A1, where it is assumed that the plates’ width *w* is larger than their spacing *h*, so for a simplified analysis of the structure the fields, it can be assumed to be the same as if the plates were of infinite width, which means that any edge effects can be neglected.

It can be shown [36,37] that the solution of Maxwell equations allows for three different classes of waves for the parallel plate transmission line: transverse electromagnetic (TEM, with $E_{x}=0$, and $H_{x}=0$), transverse electric (TE, with $E_{x}=0$) and transverse magnetic (TM, with $H_{x}=0$). Below the cut-off frequencies of the TE and TM modes, it can be assumed that between the parallel plates there is only the TEM mode; thus, in the sinusoidal steady state, the field components are:

(A1) $$E_{y}\left(x\right)=E_{0}e^{\mp jkx}$$

(A2) $$\eta H_{z}\left(x\right)=\pm E_{0}e^{\mp jkx}$$

where $\eta =\sqrt{\mu /\epsilon }$ and $k=\omega \sqrt{\mu \varepsilon }$ and (A1) and (A2) represent both progressive and regressive waves, whose ratio depends on the load connected at the end of the line (*x = L*).

Because $H_{x}=0$, the voltage between the two plates can be calculated by integrating $E_{y}$ along any path drawn between them:

(A3) $$V\left(x\right)=-\int_{0}^{h}E_{y}\left(x\right)dy=-hE_{0}e^{\mp jkx}$$

By applying the relationship $\vec{J}=\hat{n}\times \vec{H}$, where $\hat{n}$ is a unit vector normal to plates, the current density on one plate can be calculated from the magnetic field as:

(A4) $$J_{x}=-H_{z}$$

Then, if the plate width is *w*, the current on one plate is:

(A5) Ix=−wHz=∓wE0ηe∓jkx

In terms of transmission line voltage and current waves, the following relationships hold:

(A6) $$V\left(x\right)=V_{0}e^{\mp j\beta x}$$

(A7) Ix=±V0Z0e∓jβx

(A8) Z0=LC  β=ωLC

The electrical parameters for a parallel plane transmission line are:

(A9) C=εwh [F/m], L=μhw [H/m]

Then, from (A8):

(A10) Z0=ηhw  β=k

If the driving frequency is so low that a wavelength is much longer than the length of the line *L*, i.e.:

(A11) βL=2πLλ ≪1

a quasi-static approach can be adopted and the following approximated relationships hold:

(A12) Eyx=Vxh 

(A13) Hzx=Ixw 

Both costs and mechanical considerations have led to the development of MWTEM lines, i.e., parallel plate lines where the conducting planes are replaced by grids of parallel wires aligned along the direction of current flow.

The theoretical analysis of this kind of structure is not simple; however, under the assumption of validity of a quasi-static approximation, an approach based on potential function calculation can be profitably employed. According to [38,39], the calculation (on the transversal section) of the electric field components of the quasi-static TEM field distribution and the line impedance of an MWTEM line can be carried out according to the procedure described below.

Figure A2 shows the transversal section of an N-wire MWTEM line above an infinite ground plane, composed of a number of parallel wires lying along two planes and where:

(A14) $$\frac{r_{0}}{a}\ll 1,\;\frac{r_{0}}{b}\ll 1,\;\frac{r_{0}}{d}\ll 1,$$

Each plate consists of N wires of radius *r_0_*, equally spaced by *d*, and the ground is at a distance *b* below the bottom wires. The wires in the plane *y = −a* are at the potential −*V*/2, and the wires in the plane *y = +a* are at the potential *+V*/2.

Under a quasi-static condition and thin wires hypothesis, the solution can be based on the superposition of the static fields generated by the parallel wires with a given charge per unit length.

The potential on the *n*th wire *u_n_* is:

(A15) $$u_{n}=p_{n1}q_{1}+p_{n2}q_{2}{+\cdots +p}_{nN}q_{N}$$

where *q_i_* is the charge on the *i*th wire and each *p_ij_*, named Maxwell’s potential coefficient, is a function of the wire locations, *p_ij_ = p_ji_* and *p_ij_* ≥ 0 for all *i,j*.

By inverting the *p_ij_* matrix (N × N), one obtains the *k_pq_* matrix (N × N), whose elements are defined as capacitance coefficients:

(A16) $$q_{n}=k_{n1}u_{1}+k_{n2}u_{2}{+\cdots +k}_{nN}u_{N}$$

where *k_rs_* is the capacitance coefficient and

*k_rs_ = k_sr_*,

*k_rs_ ≥* 0 for *r = s*

*k_rs_ ≤* 0 for *r ≠ s*.

By assuming the same potential on all the N wires:

(A17) $$u_{1}=u_{2}=\cdots =u_{N}=u_{0}$$

According to (A16), the charge on the *n*th wire is:

(A18) $$q_{n}=u_{0}\sum_{m=1}^{N}k_{nm}$$

If, according to Figure A2, the transmission line is composed of N pairs of thin wires above a perfectly conducting ground, it is possible to show that [38,39]:

(A19) $$p_{nm}=\frac{1}{2}\ln\left[\frac{{\left[{\left(n-m\right)}^{2}d^{2}+4a^{2}\right]}^{2}}{{\left(n-m\right)}^{4}d^{4}+4{\left(n-m\right)}^{2}a^{2}d^{2}}\right]-\frac{1}{2}\ln\left[1+\frac{a^{2}}{{\left[b+\left(\frac{n+m-2}{2}\right)d\right]}^{2}}\right]\;\;\mathrm{for}\;m\;\neq \;n$$

(A20) $$p_{nn}=\ln\left[\frac{2a}{r_{0}}\right]-\frac{1}{2}\ln\left[1+\frac{a^{2}}{{\left[b+\left(\frac{n-1}{2}\right)d\right]}^{2}}\right]\;\;\mathrm{for}\;m\;=\;n$$

By applying a numerical inversion technique to the *p_ij_* matrix (N × N), it is possible to find the *k_pq_* matrix (N × N) and then define and calculate the effective plate separation *a_eff_* as:

(A21) 1aeff=∑n=1N∑m=1Nknm2a1+N−1d2a−n−1da2−2a1+N−1d2−n−1d+2b2

If one defines an electric field efficiency factor

(A22) $$f_{E}=\frac{a}{a_{eff}}$$

it is then possible to calculate the y component of the electric field in the center of the transversal section of the line according to the relationship:

(A23) $${E_{y}}_{\left|\begin{array}{c} y=0\\ z=\frac{\left(N-1\right)}{2}d \end{array}\right.}=\frac{\Delta V}{{2a}_{eff}}=\frac{\Delta V}{2a}f_{E}$$

where Δ*V* is the difference of potential across the two plates of the line.

The transversal electric field components *E_y rel_*, *E_z rel_* (normalized to the field strength in the *y*-direction at the center of the line) can be calculated according to the following relationships:

(A24) $$\begin{array}{cc} E_{y_{rel}}\left(y,z\right)= & \frac{E_{y}}{{E_{y}}_{\left|\begin{array}{c} y=0\\ z=\frac{\left(N-1\right)}{2}d \end{array}\right.}}=\frac{a}{f_{E}}\sum_{n=1}^{N}\left\{\left(\sum_{m=1}^{N}k_{nm}\right),\left[\frac{y+a}{{\left(y+a\right)}^{2}+{\left[z-\left(n-1\right)d\right]}^{2}}-\frac{y-a}{{\left(y-a\right)}^{2}+{\left[z+\left(n-1\right)d\right]}^{2}}\right],+\right.\\ & \left.\left(\sum_{m=1}^{N}k_{nm}\right)|\left[\frac{y-a}{{\left(y-a\right)}^{2}+{\left[z+\left(n-1\right)d+2b\right]}^{2}}-\frac{y+a}{{\left(y+a\right)}^{2}+{\left[z+\left(n-1\right)d+2b\right]}^{2}}\right]\right\} \end{array}.$$

(A25) $$\begin{array}{cc} E_{z_{rel}}\left(y,z\right)= & \frac{E_{z}}{{E_{y}}_{\left|\begin{array}{c} y=0\\ z=\frac{\left(N-1\right)}{2}d \end{array}\right.}}=\frac{a}{f_{E}}\sum_{n=1}^{N}\left\{\left(\sum_{m=1}^{N}k_{nm}\right)\left[\frac{z-\left(n-1\right)d}{{\left(y+a\right)}^{2}+{\left[z-\left(n-1\right)d\right]}^{2}}-\frac{z-\left(n-1\right)d}{{\left(y-a\right)}^{2}+{\left[z-\left(n-1\right)d\right]}^{2}}\right]-\right.\\ & \left.\left(\sum_{m=1}^{N}k_{nm}\right)|\left[\frac{z+\left(n-1\right)d+2b}{{\left(y+a\right)}^{2}+{\left[z+\left(n-1\right)d+2b\right]}^{2}}-\frac{z+\left(n-1\right)d+2b}{{\left(y-a\right)}^{2}+{\left[z+\left(n-1\right)d+2b\right]}^{2}}\right]\right\} \end{array}$$

Under the assumption of the existence of only a TEM mode, the impedance of the transmission line is related to the free-space characteristic impedance by the following relationship [39]:

(A26) $$Z=f_{g}\sqrt{\frac{\mu_{0}}{\varepsilon_{0}}}≅120\pi f_{g}$$

where *f_g_*, defined as geometrical factor, is:

(A27) $$f_{g}=\frac{u_{1}}{\pi \sum_{n=1}^{N}\left(\sum_{m=1}^{N}k_{nm}\right)}$$

and where:

(A28) $$u_{1}=\left(\sum_{m=1}^{N}k_{1m}\right)ln\left(\frac{2a}{r_{0}}\right)+\frac{1}{2}\sum_{n=2}^{N}\left\{ln\left[\frac{{\left[{\left(n-1\right)}^{2}d^{2}+4a^{2}\right]}^{2}}{{\left(n-1\right)}^{4}d^{4}+4{\left(n-1\right)}^{2}a^{2}d^{2}}\right]\left(\sum_{m=1}^{N}k_{nm}\right)\right\}\;-\frac{1}{2}\sum_{n=1}^{N}\left\{ln\left[1+\frac{a^{2}}{{\left[b+\left(\frac{n-1}{2}\right)d\right]}^{2}}\right]\left(\sum_{m=1}^{N}k_{nm}\right)\right\}$$

By substituting in (A19), (A20), (A27) and (A28) the geometrical parameters of the MWTEM line used for the experimental activities described in the paper, i.e., *N* = 7 (number of wires per plate), *d* = 0.25 m (wire spacing), *r_0_* = 0.01 m (wire radius), *a* = 1.16 m (half the distance between the wire plates), and *b* = 0.8 m (distance between the bottom wires and the ground), it is possible to calculate the geometrical factor (*f_g_* = 0.5511) and then *Z* ≅ 120 π *f_g_* = 207.75 Ω, which is in good accordance with the value of the MWTEM line load (200 Ω, value equal to the line impedance, according to the common criterion used for a transmission line load selection).

## Appendix B

Electric and magnetic field integral equations (EFIE and MFIE) have the following general forms:

(A29) $$\vec{E}=f_{e}\left(\vec{J}\right),\;(\mathrm{EFIE})$$

(A30) $$\vec{H}=f_{m}\left(\vec{J}\right),\;(\mathrm{MFIE})$$

where $\vec{E}$ and $\vec{H}$ are the electric and magnetic field vectors and the currents $\vec{J}$ are the field sources, which in the method of moments (MoM) [32] formulation are expressed as a sum of basis function:

(A31) $$\vec{J}=\sum_{i=1}^{M}J_{i}{\vec{b}}_{i}$$

where ${\vec{b}}_{i}$ are the basis functions and $J_{i}$ their coefficients.

For a general problem of determining the electromagnetic field generated by currents flowing on a conductive structure, some current contributions are known a priori (i.e., forced by sources), while others (on both the same structure and different ones) are the unknowns of the problem. Once the currents have been calculated, the electric and magnetic vector fields can be determined, both in the near field and in the far field.

The electric and magnetic field integral equations that have to be solved take the following form [29]:

(A32) $$F\left(g\left(\vec{r}\prime \right)\right)=h$$

where *F* is a linear operator, *h* is a known excitation function, and *g* is the system response to be found. According to the MoM formulation, the unknown function *g* is expressed as a linear combination of N terms:

(A33) $$g\left(\vec{r}\prime \right)\approx a_{1}g_{1}\left(\vec{r}\prime \right)+a_{2}g_{2}\left(\vec{r}\prime \right)+\cdots +a_{N}g_{N}\left(\vec{r}\prime \right)=\sum_{n=1}^{N}a_{n}g_{n}\left(\vec{r}\prime \right)$$

where $a_{n}$ are unknown constants and $g_{n}\left(\vec{r}\prime \right)$ are known functions, namely the basis functions, belonging to the same domain of $g\left(\vec{r}\prime \right)$. Then, (A32) can be written as:

(A34) $$\sum_{n=1}^{N}a_{n}F\left(g\left(\vec{r}\prime \right)\right)=h$$

The solution of (A34) is based on the linearity of the operator; by expanding the expression in N linearly independent equations, imposing the boundary conditions in N different points by means of a point-matching technique and employing an inner product is defined as:

(A35) $$<w,g>=\int_{S}w^{*}gds$$

where *S* is the surface of the structure under analysis and *w* is the test function, defined in the same domain of the *F* function (usually a Dirac function):

(A36) $$\left[w_{m}\right]=\left[\delta \left(\vec{r}-{\vec{r}}_{m}\right)\right]\;\;\;\;\;m\;=\;1,\;2,\;\ldots ,\;N$$

In (A36), $\vec{r}$ is a given position in the reference coordinate system and ${\vec{r}}_{m}$ is the position of application of the boundary conditions.

By applying (A35) and (A36) in (A34), the following relationship is obtained:

(A37) $$\sum_{n=1}^{N}a_{n}<w_{m},F(g_{n})>=<w_{m},h>\;\;\;\;\;m\;=\;1,\;2,\;\ldots ,\;N$$

which is written in the matrix form and solved for the unknown coefficients by means of a matrix inversion operation, which leads to:

(A38) $$\left[a_{n}\right]={\left[G_{mn}\right]}^{-1}\left[h_{m}\right]$$

where:

(A39) $$\left[G_{mn}\right]=\left[\begin{array}{ccc} <w_{1},F(g_{1})> & <w_{1},F(g_{2})> & \cdots \\ <w_{2},F(g_{1})> & <w_{2},F(g_{2})> & \\ \vdots & & \ddots \end{array}\right],\;\left[a_{n}\right]=\left[\begin{array}{c} a_{1}\\ a_{2}\\ \vdots \\ a_{N} \end{array}\right]\;e\;\left[h_{m}\right]=\left[\begin{array}{c} <w_{1},h>\\ <w_{2},h>\\ \vdots \\ <w_{N},h> \end{array}\right]$$

In the case of structures composed of conductive filamentary elements (wires), for the Numerical Electromagnetics Code (NEC), the unknown function $g\left(\vec{r}\prime \right)$ is the linear current $I\left(s\right)$ that flows along the elements. According to the MoM, each conductive element is assumed to be subdivided in segments, and on each of them, the current $I\left(s\right)$ is expanded in the following form [29]:

(A40) $$I_{i}(s)=A_{i}+B_{i}\sin k(s-s_{i})+C_{i}\cos k(s-s_{i}),\;\mathrm{dove}\;\left|s-s_{i}\right|<\Delta_{i}/2$$

where s is the position coordinate along the segment of length Δ and $s_{i}$ is the value of s in the center of the segment. As discussed in the literature [40,41], using this kind of form guarantees a rapid convergence of the solution.

## Appendix C

A detailed numerical analysis of the electric and magnetic field components was carried out for a thorough understanding of the characteristics of the field generated inside the volume formed by the MWTEM transmission line. The field generated inside the MWTEM volume is at an electrical short distance from the sources, and the properties of a far-field TEM mode cannot be a priori assumed.

According to the international standard IEC 61000-4-20 [42], which specifies the characteristics of TEM waveguides both for the generation and measurement of electromagnetic fields during their use for electromagnetic compatibility activities, a TEM is a mode in which the components of the electric and magnetic fields in the propagation direction ($E_{x}$ and $H_{x}$ in the case of interest) are much less than the primary field components ($E_{y}$ and $H_{z}$) across any transverse cross-section. The TEM mode is considered to be dominant if statistically at least 75% of the secondary (unintended) electric field components are at least 6 dB less than the primary component of the electric field. Given the good accordance of simulations and measurements observed during the MWTEM validation activity, the TEM mode verification was carried out by simulation by using the numerical model described in the paper, using the input voltages and the load impedance values of Table 2.

With reference to the geometry of the line shown in Figure 3, Figure 7 and Figure 8, a transversal and a longitudinal section of 1 × 1 m were defined, and on them, the *x*, *y* and *z* components of the electric and magnetic field vectors were calculated and analyzed. The results for the transversal section for four frequency values (0.3 MHz, 5 MHz, 10 MHz, and 20 MHz) are shown in Figure A3 and Figure A4 for the electric field and magnetic vector components, respectively, while the same kind of results for the longitudinal section are shown in Figure A5 and Figure A6. Even if the primary requirement was on the electric field (to which the field probes of interest are sensitive), in view of using the MWTEM line also for magnetic field probe calibration, the analysis was also carried out on the magnetic field vector components.

As can be observed in Figure A3, Figure A4, Figure A5 and Figure A6, the primary field components $E_{y}$ and $H_{\;z}$ are always greater than the secondary ones ($E_{x}$, $E_{z}$, $H_{x}$, $H_{y}$). All of the simulated results obtained on the transversal and longitudinal planes were analysed in detail, and the differences (in dB) between the primary and the secondary components are reported in Table A1. All of the secondary components are 6 dB less than the primary components, both for the electric and the magnetic field vectors. Moreover, the transversal components $E_{z}$ and $H_{y}$, orthogonal to the principal ones ($E_{y}$, $H_{z}$), are at least 15 dB below, thus confirming a uniform linear polarization. Similar results can be hypothesized in all of the 0.3–20 MHz frequency band.

**Table A1:** MWTEM transmission line—primary and secondary field vector components analysis.

Freq. [MHz]	Transversal Section (x = 0, −0.5 < y < 0.5, 1.05 < z < 2.05)				Longitudinal Section (−0.5 < x < 0.5, y = 0, 1.05 < z < 2.05)			
	EyEx [dB]	EyEz [dB]	HzHx [dB]	HzHy [dB]	EyEx [dB]	EyEz [dB]	HzHx [dB]	HzHy [dB]
0.3	>40	15.5	>40	18.7	>40	>40	23.9	>40
5	>40	15.4	35.3	18.8	>40	>40	23.1	>40
10	38.8	15.2	32.9	18.9	>40	>40	23.7	>40
20	>40	14.6	17.9	18.4	>40	>40	15.1	>40

For the sake of completeness, the wave impedance has also been calculated along two transversal paths (x = 0, −0.5 < y < 0.5, z = 1.55 m and x = 0, y = 0, 1.05 < z < 2.05) and on a longitudinal path (−0.5 < x < 0.5, y = 0, z = 1.55 m). The results are shown in Figure A7 and Figure A8, where impedance values varying around the free space impedance $Z_{0}=120\;\pi$ Ω can be observed. It is worth noting that the free space impedance could be observed only in a line having an electrical length sufficient to evidence progressive and regressive waves; because of the dimensions of the transversal section of the MWTEM line, this condition would occur for frequency values at which non-negligible field perturbations due to high-order modes would arise.
